# Novel Method of Non-contact Remote Measurement of Neuronal Electrical Activity

**DOI:** 10.7759/cureus.3384

**Published:** 2018-09-28

**Authors:** Tyler A Carson, Hammad Ghanchi, Harjyot Toor, Gohar Majeed, James G Wiginton, Yongming Zhang, Dan E Miulli

**Affiliations:** 1 Neurosurgery, Riverside University Health System, Riverside, USA; 2 Neurosurgery, Riverside University Health System, Moreno Valley, USA; 3 Neurosurgery, Riverside University Health System, Colton, USA; 4 Medical Physics, Quasar Federal Systems, San Diego, USA

**Keywords:** electromagnetic field, brain imaging, non-invasive imaging, neuronal stimulation

## Abstract

Measuring the electrical potential of a neuron cell currently requires direct contact with the cell surface. This method requires invasive probing and is limited by the deflection of electricity from baseline. From a clinical perspective, the electrical potential of the brain's surface can only be measured to a depth of one centimeter using an electroencephalogram (EEG), however, it cannot measure much deeper structures. In this trial, we attempt a novel method to remotely record the electromagnetic field (EMF) of action potential provoked from hippocampal neurons without contact.

A bipolar stimulating electrode was placed in contact with the CA1 region of viable hippocampal slice from donor mice. The specimen was bathed in artifical cerebrospinal fluid (aCSF) to simulate in vivo conditions. This setup was then placed into a magnetic shielded tube. Very low-frequency EMF sensors were used to obtain recordings. The impedance of the aCSF and hippocampal slice were measured after each stimulation individually and in combination.

An electromagnetic signal was detected in three out of four scenarios: (a) aCSF alone with electrical stimulus without a hippocampal slice, (b) Hippocampal slice in aCSF without electrical stimulus and, (c) Hippocampal slice in aCSF with an electric stimulus applied. Therefore, our trial suggests that EMFs from neuronal tissue can be recorded through non-invasive non-contact sensors.

## Introduction

Neurological conditions, such as brain tumors, learning disorders, Alzheimer’s disease, Parkinson’s disease, and traumatic brain injury, have debilitating clinical presentations and profound social, financial, and emotional burden. Neurochemical alterations in these diseases may be measured through blood or cerebrospinal fluid testing. It is difficult to measure the precise electrical neurophysiology of the tissue in these diseased states, therefore mainstay treatment involves a trial of pharmacology, medicine, and surgery and largely functions under the realm of supportive care, many times leaving these conditions disabling. The introduction of pulsed electrical magnet field therapy (PEMF), transcranial electrical stimulation (TES), and transcranial magnetic stimulation (TMS) offers new treatments and insight into a previously thought static pathology revealing that injured neurons remain dynamic, though have altered electromagnetic characteristics [1-6].

The neurophysiology of the neuron can be approximated through the electrical potential or electromagnetic field (EMF). Measuring the electrical potential of a neuron and dendrite requires invasive probes contacting the cell surface. This method of investigation, besides requiring direct contact, also reports only limited information as the deflection of electricity from baseline. From a clinical perspective, the electrical potential of the brain's surface can only be measured to a depth of one centimeter using a direct contact recording as in an electroencephalogram (EEG) [7]. This consists of approximately 10,000 neurons per cubic millimeter (mm) [8]. Although important information can be determined from the EEG, it cannot measure much deeper structures and it cannot tell the characteristics of the electrical information produced from the signal.

Magnetoencephalography (MEG) and magnetic resonance imaging (MRI) measure the EMF of the brain or cellular components producing an image of function. Functional MRI measures focal areas of cerebral blood flow and assumes neuronal activity is coupled to blood flow. Unfortunately, MEG and MRI machines are large and expensive, and not designed for continuous field measurement. Neuroscientists have attempted to adapt this limited information to develop current therapeutic strategies to alter neuronal [9-18] and brain [19-27] function. However, without a more extensive understanding of neurons, which includes an understanding of their EMF signal, therapeutic strategies can only be approximated through trial and error.

In this trial, we attempt a novel method to remotely record the electromagnetic field of action potentials provoked from hippocampal neurons without contact.

## Materials and methods

Testing was performed on fresh hippocampal slices supplied by our Institutional Animal Care and Use Committee (IACUC) certified neuroscience research lab from eight donor neonatal mice. Each specimen measured approximately 5 mm by 5 mm by 300 microns thick and were immediately submerged into a brain slice chamber containing artificial cerebrospinal fluid (aCSF) that was continuously circulated to replenish the nutrients and ensure viability. Artificial CSF was created by combining one liter of sterile water with 0.15 grams (g) NaH_2_PO_4_, 7.246g NaCl, 0.2236g KCl, 0.308g CaCl_2_, 0.1565g MgSO_4_, 2.184g NaHCO_3_, and 1.8 g glucose.

A single bipolar electrode was placed in contact with the Cornu Ammonis 1 (CA1) region of the hippocampal slice, whose viability was verified under microscopic illumination prior to electrode placement. The brain slice chamber (Scientific Systems Design Inc. Ontario, Canada) was warmed to 32˚C and supplied with a mixture of 5% carbon dioxide and 95% oxygen to maintain neuronal tissue viability. This setup was then placed into a zero-gauss triple magnetic shielded tube. The magnetic shield composition consists of nickel, molybdenum, silicon, manganese, carbon, and iron (MuMetal Magnetic Shield Corporation, Bensenville, IL). Very low-frequency EMF sensors were mounted at 0, 180, and offset 45 degrees, 3 centimeters (cm) away from the longitudinal axis of the hippocampal slice; sensors were BS-1000 magnetic sensors (Quasar Federal Systems, San Diego, CA), at gain setting F, with a 10x gain/2 kilohertz (kHz) gain/filter module, ultra-low noise magnetic induction sensors 1 Telsa per square root Hertz (pT/rtHz), ultra-low frequency band 1 Hertz (Hz). The impedance of the aCSF and hippocampal slice were measured individually and together. After each stimulation, the impedance of the hippocampal slice was again measured. The electromagnetic signals were measured in the following combinations: 1) aCSF alone without electric stimulus, 2) aCSF alone with electrical stimulus via bipolar electrode, 3) Hippocampal slice in aCSF without electrical stimulus and, 4) Hippocampal slice in aCSF with electric stimulus applied (Table 1).

**Table 1:** Combination of Tested Scenarios The four different setups tested are demonstrated in the table above with the result in last column. Abbreviations: aCSF – Artificial Cerebrospinal Fluid, EMF – Electromagnetic Field

	aCSF	Stimulus	Hippocampal Slice	EMF Signal Detected
Combination 1	+	-	-	-
Combination 2	+	+	-	+
Combination 3	+	-	+	+
Combination 4	+	+	+	+

Stimulation was performed from 1-10 Hertz (Hz), 10 millivolts (mV) - 5 volts (V), pulse width (PW) 0.1- 100 miliseconds (ms), and 10 - 60 seconds of stimulation followed by 20-300 seconds of no stimulation. Some samples were measured after stimulation until tissue death. The sensor output was captured on Microsoft Windows Platform using a National Instrument (NI) 16-bit data acquisition (DAQ) module, sampling at 5 kilosamples per second (kS/s), voltage range +/- 0.5V (G =50), and the collected data was analyzed using Igor Pro version 6 (WaveMetrics, Inc. Portland, OR). Four different hippocampal slices from each of eight different mice were analyzed in over 600 recordings.

## Results

There was no electromagnetic signal obtained from the chamber with aCSF alone and no stimulus (Table 1). An electromagnetic signal was detected from the latter three samples: aCSF alone with electrical stimulus via a pair of bipolar electrodes, hippocampal slice in aCSF without an electrical stimulus, and hippocampal slice in aCSF with an electric stimulus applied.

**Figure 1:**
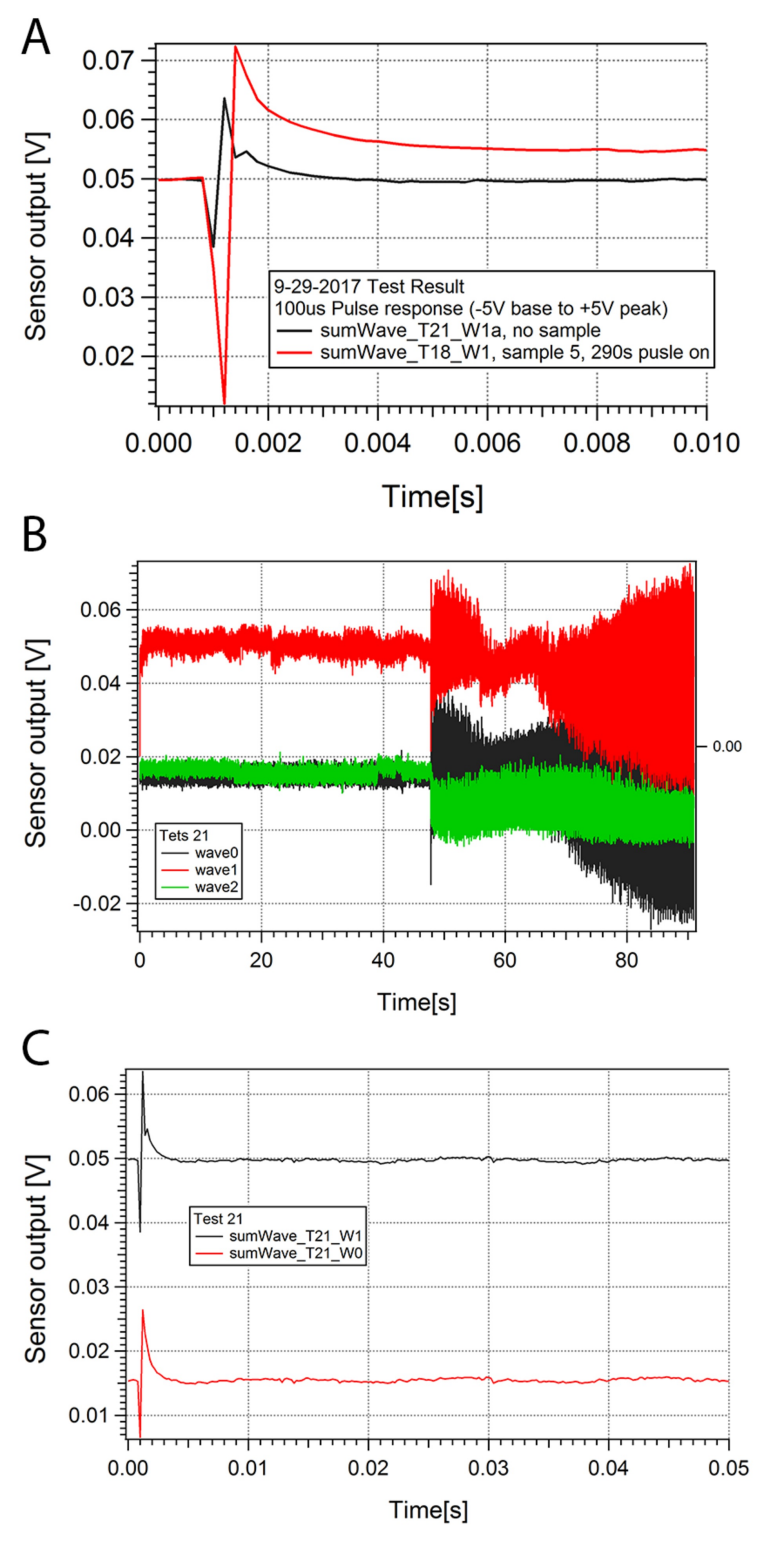
Stimulation of Artificial CSF with and without Hippocampal Slice Sample Figure 1A demonstrates stimulated sensor response for sensor 1 (averaged over 100 pulses) without sample (black curve) and with sample (red curve) to illustrate the difference in amplitude; Figure 1B shows responses for three sensors (sensor 0 - black, sensor 1 - red, sensor 2 - green) without sample, with stimulation turned on at time = 50 seconds; Figure 1C demonstrates stimulated response (averaged for 100 pulses) for sensor 0 (red curve) and 1 (black) with no sample. Abbreviations: V - Volts, s - Seconds

**Figure 2:**
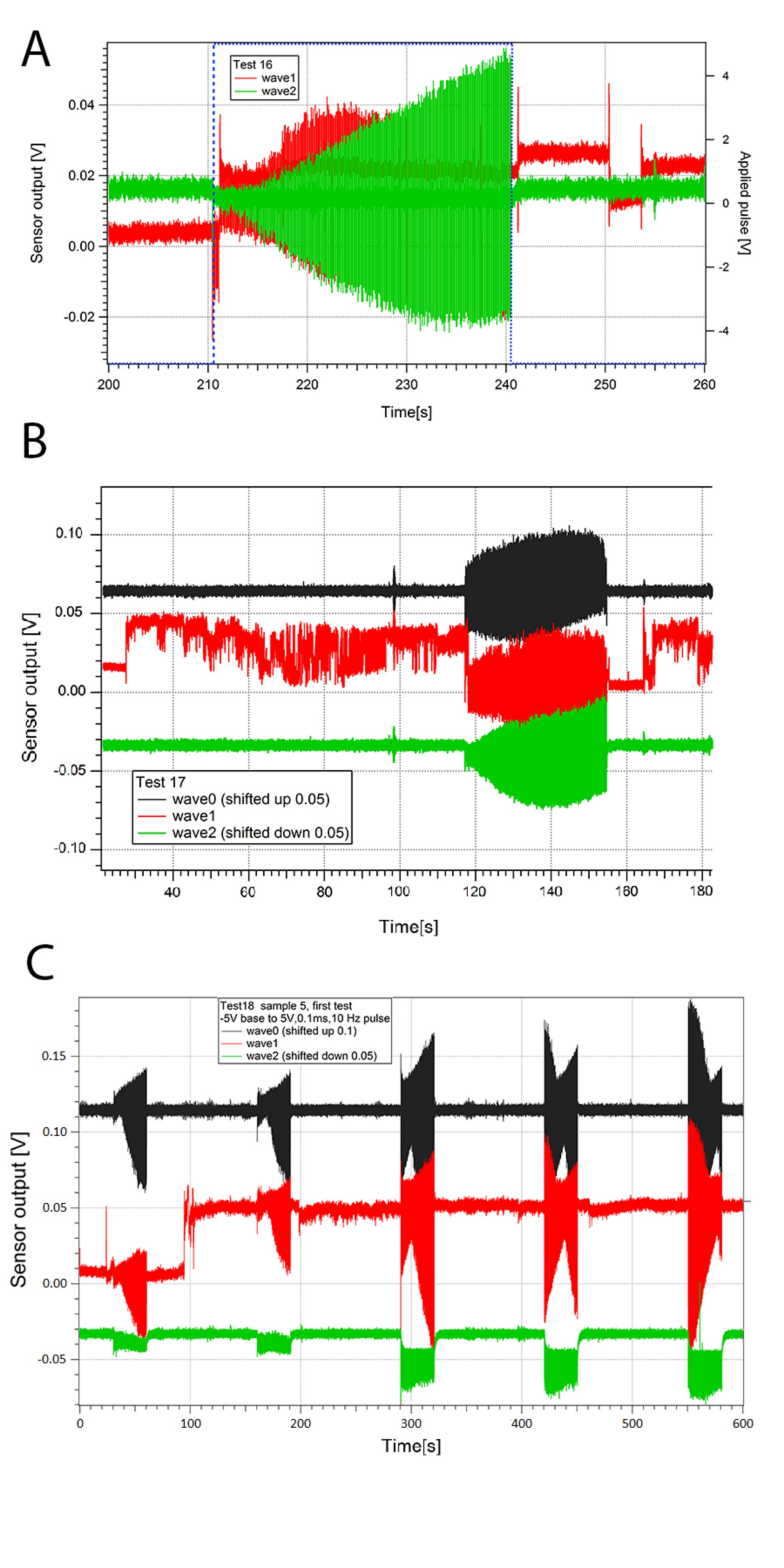
Stimulation of Artificial CSF and Hippocampal Slice Figure 2A demonstrates Sensor 1 (wave 1) and 2 (wave 2) responses with stimulation (10V peak-to-peak, 100 ms pulse width, 10 Hz repeat rate) turned on between 210.5 second to 240.5 second. Figure 2B shows continued measurement of Figure 2A. Stimulation turned on again from 118 second to 147 second. Some DC level changes were observed on sensor 1 (wave 1). Figure 2C demonstrates sensor responses with stimulation on and off. A noticeable DC shift was observed on sensor 1 at 100s. Abbreviations DC - Direct Current, V - Volts, s - Seconds

**Figure 3:**
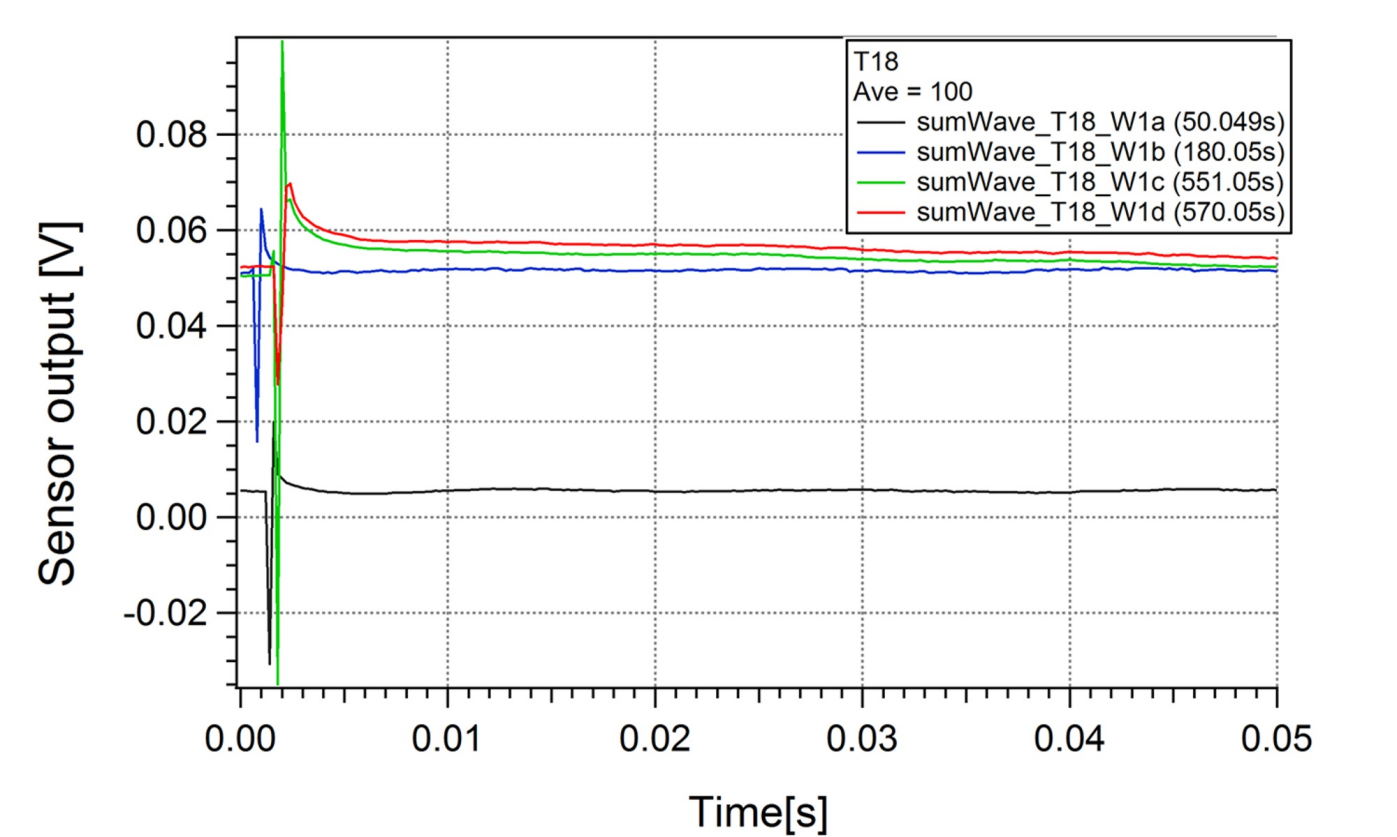
Stimulated Sensor 1 Responses Stimulated sensor responses for sensor 1 at different times. Looking carefully, the stimulated waveforms at later times (551.05 s for green curve and 570 s for red curve) are very different from stimulated waveforms at early times (50.049 seconds for black, 180.05 seconds for blue). Later waveforms have a long decay time than early waveforms. This observation may indicate that it will need a certain amount pulses to change/stimulate the electromagnetic state of the sample. Abbreviations: V - Volts, s - Seconds

**Figure 4:**
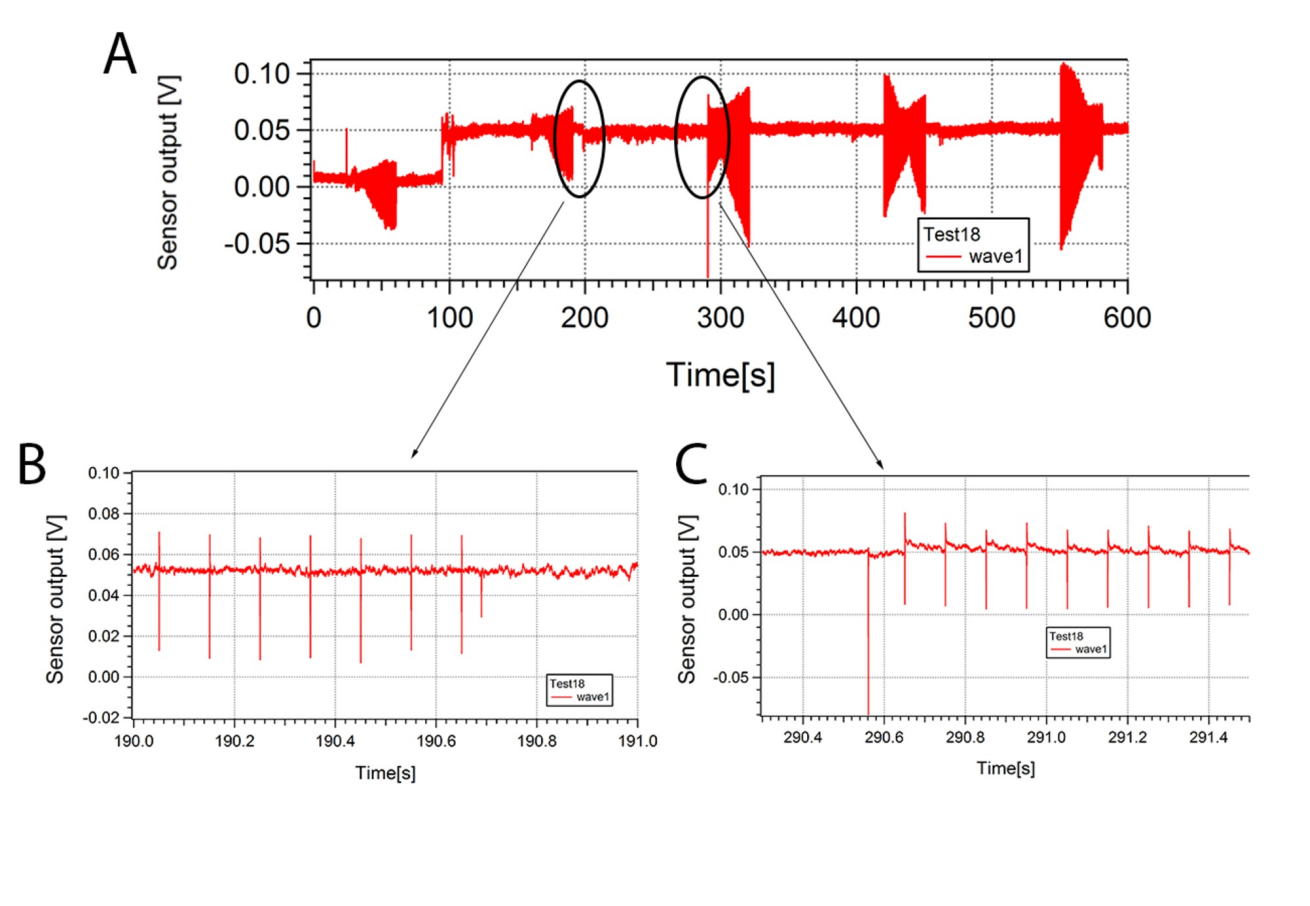
Change of Electromagnetic State Figure 4A demonstrates stimulated response for sensor 1 with hippocampal slice with focus between Figure 4B and Figure 4C; Figure 4B is a zoom-in of the response around 190 seconds, and Figure 4C is a zoom-in of the response around 290 seconds. The response changed from quick recovery (Figure 4B) to slow recovery (Figure 4C), indicating a change of electromagnetic state in the sample. Abbreviations: V - Volts, s - Seconds

The aCSF alone generated a specific waveform when stimulated without a sample (Figure 1). This was likely secondary to the capacitance of aCSF between bipolar electrodes. This waveform, however, was different from the waveform obtained with stimulation of the hippocampal slice within aCSF (Figures 1A, 2-4).

**Figure 5:**
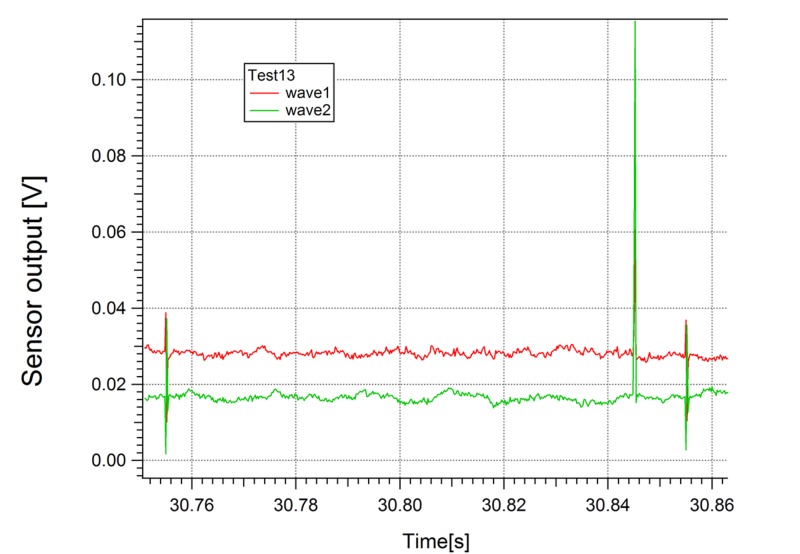
Non-stimulated Hippocampal Slice Abbreviations: V - Volts, s - Seconds

A waveform (Figure 5) was recorded in the unstimulated hippocampal slice but could not be qualified or quantified to determine validity or reproducibility because no action potential took place in the tissue container, or the signals were below the detection sensitivity of the sensors. The EMF signal released corresponded in the three sensors, was opposite in polarity in wave 1 (sensor 1) and wave 2 (sensor 2), and was much lower amplitude than with the stimulated action potential. The hippocampal stimulation that produced the most reliable results were 10 Hz, 5 V peak to peak, 0.1 ms PW, 20 seconds on, 20 seconds off.

A signal was identified from the hippocampal slice being stimulated with a pair of bipolar electrodes (Figure 2); these waveforms are different from aCSF alone (Figure 1). The combined waveform appeared to match the signature and wavelength of waveforms obtained from direct recordings of the hippocampal slice via a contact electrode (Figure 1). With aCSF and hippocampal slice, the average waveform changes with and without the stimulus, which is different from aCSF alone. The waveforms consisted of many individual waves when the signal time-scale was expanded and analyzed. The reproducible hippocampal slice waveform sensed from each probe corresponded to the time (pulse width and time between pulses), size (pulse amplitude), characteristics (pulse shape) of the stimulus, and the orientation of the sensor. The entire waveform direct current (DC) levels changed with the on stimulus in the appropriate deflection up or down depending upon the location of the sensor. The shift in DC recovered after the stimulus was off and deflected again with further stimulus. When an individual wave is analyzed there is a slow decay in the response back to baseline only seen with a hippocampal slice (Figure 3). The individual waveforms from the hippocampal slices are unique to the sample and stimulation in the degree of deflection from DC, decay to baseline, and minor changes in shape (Figure 2 and Figure 4). Some hippocampal slices were stimulated until there was no change from the DC baseline, no slow decay to baseline, and no unique waveform. At that time the impedance of the tissue was found to be at least ten times the initial impedance and upon microscopic inspection was found to not be viable.

The waveform obtained from stimulating aCSF without a sample still showed a down shift as seen on wave 2 with the stimulus on (Figure 1B). Changing the DC current did not reliably predict a wave amplitude response. When the averaged wave 0 is compared to the averaged wave 1, the exact same waveform is seen with the exception of having a slightly larger averaged wave amplitude in wave 1 (Figure 1C). Additionally, without a sample the wave 0 response is bipolar, and recovered within 50 microseconds; however, with a stimulated sample, wave 1 response is unipolar and it takes more than 100 milliseconds to recover (Figure 1A).

## Discussion

The human nervous system is an electrical circuit; electricity flow is generated from complex interaction of ions, proteins, and the inherent architecture of neurons. This electrical flow makes up the basis of neurophysiology. According to Peters, Hendriks, and Stinstra [28], the electrical conductivity of human tissue containing many cells at low frequencies in a uniform electric field can be described as a suspension of particles in a conducting solution and considers the cell as a non-conducting particle. Usually, a tissue is composed of several types of particles. A relationship that expresses the effective conductivity of a suspension of one type of ellipsoidal particles could be found in the literature [28]. The orientation of the particles could be uniform or they could be randomly distributed and is measured during this procedure.

However, a less studied phenomenon is the EMF emitted by neuronal tissue. Furthermore, no mainstream clinical applications employ these signals. These signals may permit the brain to synchronize various regions to perform complex procedures. These signals can travel from one part of the brain to another without a physical medium, much like a radio or cell phone receives its signal from a central emitting tower [29]. This was proposed and verified by Ahissar et. al. demonstrated in the 1997 Proceedings of the National Academy of Sciences [30] that they found that certain circuits in the brain work on the same principle as an FM radio. These connections are not visible through anatomical studies as no physical structure exists. The next step will be to exploit the EMF signals for therapeutic uses.

Neuronal electromagnetic signals can be measured through non-contact solid-state sensors that record very low magnitude magnetic fields at a distance. In addition, these sensors can distinguish between the background stimulated milieu, the neuron activity, and the environment. Although the EMF was recorded without contact from the non-stimulated neuronal tissue, the signal was low amplitude and could not be reliably qualified or quantified without additional filtering equipment for the purpose of this initial proof of concept. Constructing an array of sensors to identify the specific waveform permitting localization and characterization of different types of neuronal signal in the in vivo model with naturally occurring action potentials would be the next step. That same model would also identify the non-depolarizing activity of the living tissue. This study serves as a proof of concept to prove these non-contact sensors can record low magnitude electromagnetic waves in a neuronal in vitro model.

This ability to measure continuous neuronal responses through non-contact sensors is the next evolution in the treatment of neurological disease. The brain has been treated with surgery to mechanically change parts of the brain and medicine to try to add a change to the chemical environment of the system in order to get the isolated desired effect. Cellular processes are influenced by bathing it with chemicals. The best treatment would be to treat the organs, cells, and organelles on their level. These changes can be caused by the modulation of the intrinsic properties of the diseased state. Of course, medication will continue to be needed to provide the substrate for reactions to take place. The electrical energy is what is necessary and what is manipulated on all levels by the neurological system. Neuromodulation utilizes and changes the innate system in fighting disease.

The evolution of non-contact sensors may be the revolution in controlling artificial or paralyzed limbs. Although the connection may not be made, thoughts will be able to control limbs that have been implanted with biosensors. Dr. Nicolelis [10] has implanted electrodes into the brain of an owl monkey; the monkey controlled a robotic arm 600 miles away. The most impressive feat was the computer program that interpreted the thoughts of the monkey and interfaced with the robotic arm. Neuromodulation permits non-invasive reading of the electrical energy of the thought process for transmission across space. The potential for real-world clinical applications brain EMF technology may be ground-breaking with further research.

## Conclusions

Our study demonstrates the potential to record very low-frequency electromagnetic fields from neuronal tissue remotely through non-contact high-sensitivity magnetic sensors. This technology has the potential to change the way we think about and treat brain and spinal cord disease and injury. The information from neuronal EMF can be studied in every instance that neuronal electrical potential has been studied and without perturbation nor invasion of the tissue. If successful, it can provide instantaneous information on structure and function which may allow us to intervene sooner. This would open a whole new frontier into the management and treatment of nervous system disease. Extensive work is still needed to make this a reality from our proof of concept study. Our ultimate goal would be to extrapolate this technology into a portable machine we can use in the clinical setting, providing real-time feedback; e.g. in the neurointensive care unit to halt a seizure before it starts, prevent hypermetabolic states or even stimulate pathways in a therapeutic fashion to strengthen neuronal connections.
